# Supplementary material for: Microstructural, Biomechanical, and In Vitro Studies of Ti-Nb-Zr Alloys Fabricated by Powder Metallurgy
Source: Materials (Basel). 2023 Jun 8;16(12):4240. doi: 10.3390/ma16124240 (PMC10302561; doi:10.3390/ma16124240)

materials

Article

Microstructural, Biomechanical, and In Vitro Studies of Ti-Nb-Zr Alloys Fabricated by Powder Metallurgy

Eyyup Murat Karakurt [1], Yuksel Cetin [2,*], Alper Incesu [3], Huseyin Demirtas [3], Mehmet Kaya [4], Yasemin Yildizhan [2], Merve Tosun [2] and Yan Huang [1,*]

[1] BCAST, Institute of Materials and Manufacturing, Brunel University London, Uxbridge, London UB8 3PH, UK; eyyupmurat.karakurt@brunel.ac.uk
[2] TUBITAK, Marmara Research Center, Life Sciences, Medical Biotechnology Unit, Gebze 41470, Kocaeli, Turkey; yasemin.yildizhan@tubitak.gov.tr (Y.Y.); mervetosun0@outlook.com (M.T.)
[3] TOBB Technical Sciences Vocational School, Karabuk University, Karabuk 78050, Turkey; alperincesu@karabuk.edu.tr (A.I.); hdemirtas@karabuk.edu.tr (H.D.)
[4] Machinery and Metal Technologies Departmant, Corlu Vocational School, Tekirdag Namik Kemal University, Corlu, Tekirdag 59830, Turkey; mehmetkaya@nku.edu.tr
* Correspondence: yuksel.cetin@tubitak.gov.tr (Y.C.); yan.huang@brunel.ac.uk (Y.H.); Tel.: +90-(262)-6773380 (Y.C.); +44-(0)-18952-66976 (Y.H.)

Abstract: This study investigated the microstructures, mechanical properties, corrosion resistances, and biocompatibility of porous Ti-xNb-10Zr (x: 10 and 20; at. %) alloys. The alloys were fabricated by powder metallurgy with two categories of porosities, i.e., 21–25% and 50–56%, respectively. The space holder technique was employed to generate the high porosities. Microstructural analysis was performed by using various methods including scanning electron microscopy, energy dispersive spectroscopy, electron backscatter diffraction, and x-ray diffraction. Corrosion resistance was assessed via electrochemical polarisation tests, while mechanical behavior was determined by uniaxial compressive tests. In vitro studies such as cell viability and proliferation, adhesion potential, and genotoxicity were examined by performing MTT assay, fibronectin adsorption, and plasmid–DNA interaction assay. The experimental results showed that the alloys had a dual-phase microstructure comprising finely dispersed acicular hcp α-Ti needles in the bcc β-Ti matrix. The ultimate compressive strength ranged from 1019 MPa to 767 MPa for alloys with 21–25% porosities and from 173 MPa to 78 MPa for alloys with 50–56% porosities. Adding a space holder agent had a more significant effect on the mechanical behaviors of the alloys compared with adding niobium. The pores were largely open and exhibited irregular shapes, with uniform size distribution, allowing for cell ingrowth. Histological analysis showed that the studied alloys met the biocompatibility criteria required for orthopedic biomaterial use.

Keywords: powder metallurgy; porosity; space holder technique; corrosion resistance; biocompatibility

Citation: Karakurt, E.M.; Cetin, Y.; Incesu, A.; Demirtas, H.; Kaya, M.; Yildizhan, Y.; Tosun, M.; Huang, Y. Microstructural, Biomechanical and In Vitro Studies of Ti-Nb-Zr Alloys Fabricated by Powder Metallurgy. *Materials* **2023**, *16*, 4240. https://doi.org/10.3390/ma16124240

Academic Editor: In-Chul Choi

Received: 30 April 2023
Revised: 2 June 2023
Accepted: 5 June 2023
Published: 8 June 2023

Copyright: © 2023 by the authors. Submitted for possible open access publication under the terms and conditions of the Creative Commons Attribution (CC BY) license (https://creativecommons.org/licenses/by/4.0/).

Supplementary Figure S1. Plasmid–DNA interaction assay for Ti-Nb-Zr based alloys. The migration pattern of plasmid DNA incubated with Ti-Nb-Zr-based alloys and TiRG4 reference materials is shown in the figure. The bands are labeled as NC: Nicked circular and SC: Supercoiled; ddH2O served as a negative control.

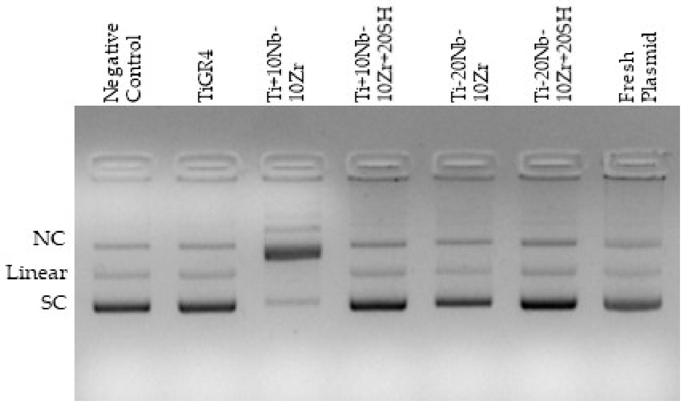

Supplement: Supplementary file 1 [file materials-16-04240-s001.zip › materials-2401094-supplementary.pdf]
